# Near-IR Electrochromic Film with High Optical Contrast and Stability Prepared by Oxidative Electropolymerization of Triphenylamine Modified Terpyridine Platinum(II) Chloride

**DOI:** 10.3390/molecules28248027

**Published:** 2023-12-09

**Authors:** Huiying Gu, Xiaomeng Sun, Qian Zhao, Hongwei Wang, Xinfeng Cheng, Chunxia Yang, Dongfang Qiu

**Affiliations:** 1 College of Chemistry, Zhengzhou University, No. 100 of Kexue Road, Zhengzhou 450001, China; 2 College of Chemistry and Pharmaceutical Engineering, Nanyang Normal University, Nanyang 473061, China

**Keywords:** terpyridine platinum(II) chloride, triphenylamine, electropolymerization, metallopolymer, near-IR electrochromism

## Abstract

Terpyridine (TPY) platinum(II) chloride with a triphenylamine (TPA) group was successfully synthesized. The strong intramolecular Donor(TPA)-Acceptor(TPY) interaction induced the low-energy absorption band, mixing the spin-allowed singlet dπ(Pt)→π*(TPY) metal-to-ligand charge transfer (MLCT) with the chloride ligand-to-metal charge transfer (LMCT) and chloride ligand-to-ligand (TPY) charge transfer (LLCT) transitions, to bathochromically shift to *λ*_max_ = 449 nm with significant enhancement and broadening effects. Using the cyclic voltammetry method, its oxidative electropolymerization (EP) films on working Pt disk and ITO electrodes were produced with tunable thickness and diffusion controlled redox behavior, which were characterized by the SEM, EDS, FT-IR, and AC impedance methods. Upon applying +1.4 V voltage, the sandwich-type electrochromic device (ECD) with ca. 290 nm thickness of the EP film exhibits a distinct color transformation from red (CIE coordinates: *L* = 50.75, *a* = 18.58, *b* = 5.69) to dark blue (CIE coordinates: *L* = 45.65, *a* = −1.35, *b* = −12.49). Good electrochromic (EC) parameters, such as a large optical contrast (Δ*T*%) of 78%, quick coloration and bleaching response times of 2.9 s and 1.1 s, high coloration and bleaching efficiencies of 278.0 and 390.5 C^−1^·cm^2^, and good cycling stability (maintains 70% of the initial Δ*T*% value after 3200 voltage switching cycles), were obtained.

## 1. Introduction

A promising class of electrochromic (EC) materials, metallopolymers derived from transition metal polypyridine complexes [1,2], have potential applications in reusable price labels, controllable display devices, environmentally adaptable smart windows, protective eyewear, and anti-glare mirrors [3,4,5]. Two prevalent strategies for creating these hybrid materials are metal ion-based supramolecular self-assembly and electrochemical methods [6,7,8]. Especially, the electropolymerization (EP) method is widely used because it provides a one-step process to avoid the sophisticated pattern steps and the solubility problems of other approaches. However, the majority of them display color variations in the visible spectrum, which are attributed to the alteration in their MLCT bands caused by M(II)/M(III) interconversion (M = Fe, Co, Ru, and Os).

Yao and Zhong et al. reported that the films generated by electrochemically reductive or oxidative EP of the cyclometalated (C^N) and (N^C^N) bis- and tri-Ru(II) complexes ((C^N) = 1,4-di(pyrid-2-yl)benzene, (N^C^N) = 1,2,4,5-tetra(2-pyridyl)benzene and 1,3,6,8-tetra(2-pyridyl)pyrene) with vinyl [9,10,11] or triphenylamine (TPA) [12,13] substituents possess near-IR spectral responses, which correspond to the characteristic intervalence charge transfer (IVCT) transition. It has been suggested in our previous work that Pt(II)-based metallopolymer films, respectively produced by oxidative EP of the neutrally charged cyclometalated (C^N^N) platinum(II) chloride [14] and its phenylacetylide [15] (C^N^N = 6-phenyl-2,2′-bipyridine), are a novel class of strong near-IR EC materials. This is likely relative to the electronic configuration of the metal center and the spatial structure of polypyridine complex. Compared to that of octahedron d^6^ Ru(II) complexes, the planar square configuration of d^8^ Pt(II) complex moieties is beneficial for electron delocalization through the whole metallopolymer backbone. To further examine the impact of the electrical effect on the near-IR EC response of polypyridine Pt(II) complexes, positively charged terpyridine (TPY) platinum(II) chloride with a TPA group was synthesized and its metallopolymers were prepared via the facilitated electropolymerization method. At the same time, the dynamic electrochromic character of the EP films in solution and in a solid-state device were mainly investigated in this paper.

## 2. Results and Discussion

### 2.1. Synthesis and Characterization

**Scheme 1:**
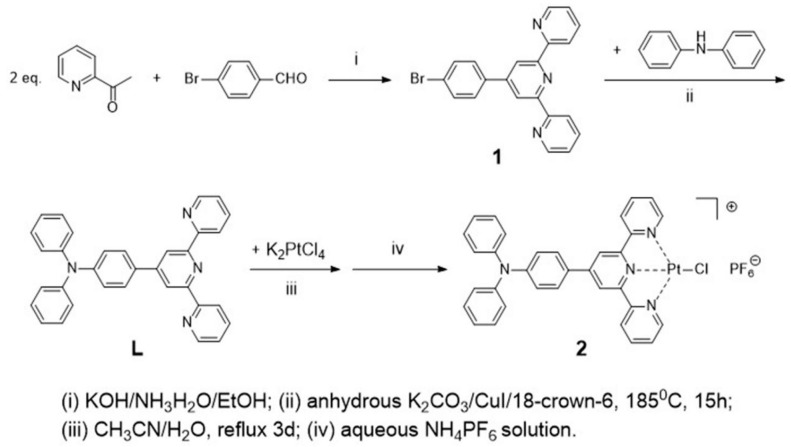
Synthetization process of the target ligand **L** and complex [(**L**)PtCl](PF_6_) (**2**).

The target ligand and its complex were synthesized following the protocol as depicted in Scheme 1. By employing commercial 2-acetylpyridine and 4-bromophenylaldehyde as the initial materials, the precursor of 4′-(4-bromophenyl)-2,2′:6′,2″-terpyridine (**1**) was produced with a yield of 86% by the prominent Kröhnke’s method. The Ullmann condensation between compound **1** and diphenylamine yielded the target ligand of 4′-[4-(diphenylamino)phenyl]-2,2′:6′,2″-terpyridine (**L**) (yield: 42%). The final Pt(II) chloride (**2**) was obtained with a yield of 65% by directly reacting **L** with K_2_PtCl_4_ salt in slight excess in a CH_3_CN/H_2_O mixed solvent, followed by precipitating the hexafluorophosphate salt and further purification on a silica gel column using CH_3_CN/sat. aqueous KNO_3_/H_2_O as the eluent. By using ^1^H NMR (Figure S1), ^13^C NMR, elemental analysis, and X-ray diffraction, their predicted structures were verified, and the crystal structure of **L** was also confirmed.

### 2.2. Crystal Structure

**Figure 1:**
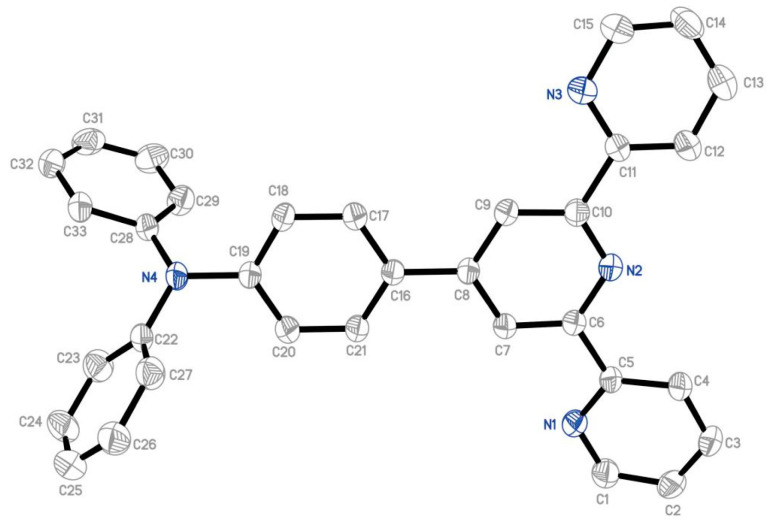
Perspective view of the crystal structure of **L** with thermal ellipsoids at 30% probability (hydrogen atoms are not presented for clarity).

Through the gradual evaporation of the CH_2_Cl_2_/MeOH solution, a single crystal of target ligand **L** (CCDC: 991875) was slowly grown. Its crystal structure and corresponding refinement parameters are shown in Figure 1 and described in Table S1, respectively. Three pyridine rings in the TPY domain display the predicted *transoid* conformation regarding the interannular C-C bonds [16]. There are 7.93 and 19.00° interplanar angles between the core pyridine ring and the two terminal pyridine rings, respectively, indicating that they are not coplanar. Intermolecular hydrogen bond formation (Figure S2) is probably responsible for this deviation from the previously reported results [17,18,19]. There is an efficient conjugation between the phenyl ring linked at position 4′ of TPY domain and the central pyridine ring, as evidenced by the interplanar angle of 2.54°. At the same time, it can be seen from the 1.434(3) Å distance of N(4)-C(19) that the N(4) atom possesses an *sp*^2^ type structure, and the amino group with the aromatic ring at 4′-position of TPY domain is strongly conjugated [20].

### 2.3. Photophysical Properties

In CH_3_CN solution, ligand **L** exhibits a high-intensity absorption peak at *λ*_max, abs_ = 289 nm (*ε* = 3.94 × 10^4^ dm^3^·mol^−1^·cm^−1^) as well as a relatively weaker absorption maximum at *λ*_max, abs_ = 358 nm (*ε* = 3.15 × 10^4^ dm^3^·mol^−1^·cm^−1^) (Figure S3) corresponding to the intraligand (IL) transition (π_TPY_-π*_TPY_) and the intramolecular charge transfer (ICT) transition (π_TPA_-π*_TPY_), respectively [21], while the [(**L**)PtCl][PF_6_] complex shows blue-shifted and weakened IL and ICT absorption bands centered at *λ*_max, abs_ = 282 nm (*ε* = 2.66 × 10^4^ dm^3^·mol^−1^·cm^−1^) and 328 nm (*ε* = 2.27 × 10^4^ dm^3^·mol^−1^·cm^−1^), respectively [22]. In addition, a broad absorption band with less intensity appears at a longer wavelength (*λ*_max, abs_ = 449 nm, *ε* = 1.54 × 10^4^ dm^3^·mol^−1^·cm^−1^), whose energy is too low for the π-π* transitions and intensity is too high for the metal-centered d-d transitions [23]. Because of the chloride ligand-to-metal charge transfer (LMCT) and chloride ligand-to-ligand (TPY) charge transfer (LLCT) transitions of the related Pt(II) complexes can also occur in this region [24], this absorption band is generally attributed to the spin-allowed singlet dπ(Pt)→π*(TPY) metal-to-ligand charge transfer (^1^MLCT) transition mixing with the LMCT and LLCT states [25]. At the same time, the incorporation of the electron-rich TPA group into the 4′-position of TPY can apparently enhance and red-shift the ^1^MLCT/LMCT/LLCT band [26]. The orbital overlapping and electron-donating effects result in its broad and intense features, compared with that of the 4′-phenyl-2,2′:6′,2″-terpyridine (PhTPY) platinum(II) chlorides [27].

### 2.4. Electropolymerization Behavior

**Figure 2:**
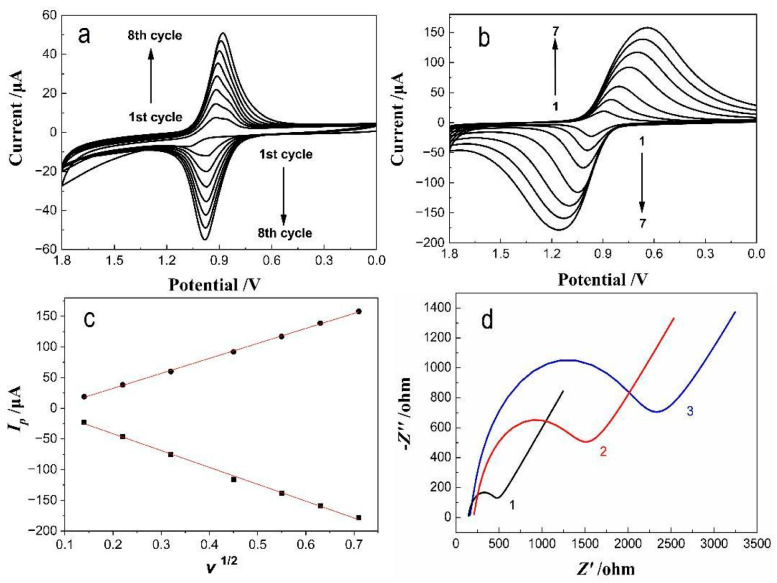
(**a**) Oxidative EP of the [(**L**)PtCl][PF_6_] complex on a Pt disk electrode at 50 mV·s^−1^; (**b**) cyclic voltammetry curves of the metallopolymer film at different potential scan rates (1: 0.02 V s^−1^; 2: 0.05 V s^−1^; 3: 0.1 V s^−1^; 4: 0.2 V s^−1^; 5: 0.3 V s^−1^; 6: 0.4 V s^−1^; 7: 0.5 V s^−1^); (**c**) the anodic/cathodic peak currents vs. the square root of the potential scan rate (*v*^1/2^); (**d**) impedance spectra of Pt disk electrodes coated with the metallopolymer film after varied CV scans (1: 10 cycles; 2: 15 cycles; 3: 20 cycles).

The electropolymerization (EP) of the [(**L**)PtCl][PF_6_] monomer onto a Pt disk electrode was performed in a 0.1 M *^n^*Bu_4_NClO_4_/CH_3_CN solution containing 0.25 mM monomer via the cyclic voltammetry (CV) method. From the cyclic voltammogram result illustrated in Figure 2a, a clear oxidation peak at *E*_p_ = +1.08 V is present in the first anodic segment, which is consistent with the one electron loss from the TPA group of **L** to form the cationic radical TPA^+^ [28]. The oxidative potential is apparently negative-shifted in the second anodic segment, and the anodic/cathodic waves steadily growing with the increase of CV scan circle demonstrate that the TPA-triggered polymerization reaction is taking place on the working electrode to produce a conductive coating with stronger electron-donating units. A compact red layer remains on the surface of the working electrode following washing with CH_3_CN to eliminate the unreacted monomer and small oligomers, and it exhibits poorly defined redox waves in electrolytic solution, particularly at rapid potential scan rates (Figure 2b). In addition, both the anodic current and the cathodic current are proportional to the square root of the potential scan rate (Figure 2c), suggesting the diffusion controlled redox process in this hybrid metallopolymer film.

Additionally, by manipulating the CV scans, a series of EP films with different thicknesses deposited onto Pt disk electrodes were produced in electrolyte solutions with a constant monomer concentration of 2.5 × 10^−4^ mol·L^−1^. The average thicknesses of the EP films obtained after 10, 15, and 20 CV cycles were determined from the SEM cross sectional analysis and found to be ca. 160, 290, and 450 nm, respectively. Their AC impedance spectra were also examined. As shown in Figure 2d, each spectrum reflects a fitted semicircle at high frequencies, whose diameter grows with the increase of the CV scan, indicating that as the film thickens, the charge transfer from the electrolyte to the working electrode surface becomes more challenging. This behavior is opposite to that of the EP film formed from neutrally charged planar (C^N^N) platinum(II) chloride [14], but is similar to those of EP films generated from the positively charged octahedron (N^N^N) and (N^C^N) Ru(II) complexes [12,13,29], suggesting that the charge migration ability is not only related to the coordination configuration of the complex core, but also to its charge number. It seems that the better the coplanarity and the smaller the charge number, the more favorable the transfer of charges within the EP film.

### 2.5. EP Film Characterization

**Figure 3:**
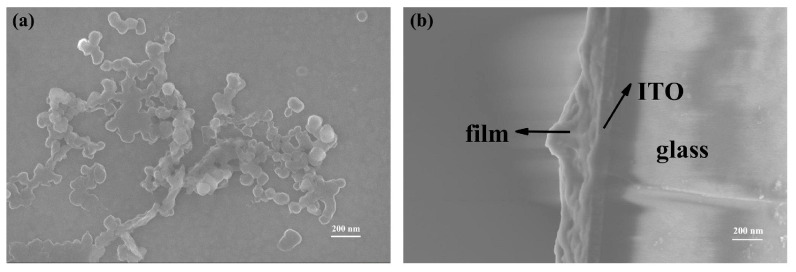
The SEM images of the surface (**a**) and the cross-sectional profile (**b**) of the EP film after 15 CV cycles.

**Figure 4:**
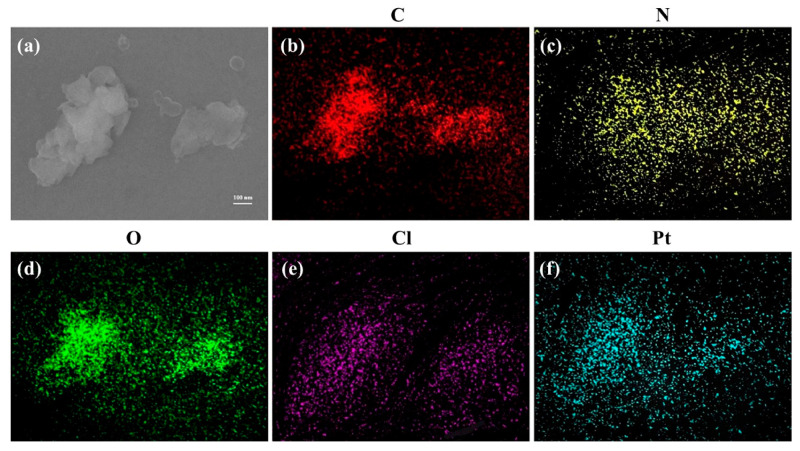
The elemental mapping mode of the EP film ((**a**) SEM image; (**b**) C element; (**c**) N element; (**d**) O element; (**e**) Cl element; (**f**) Pt element).

**Figure 5:**
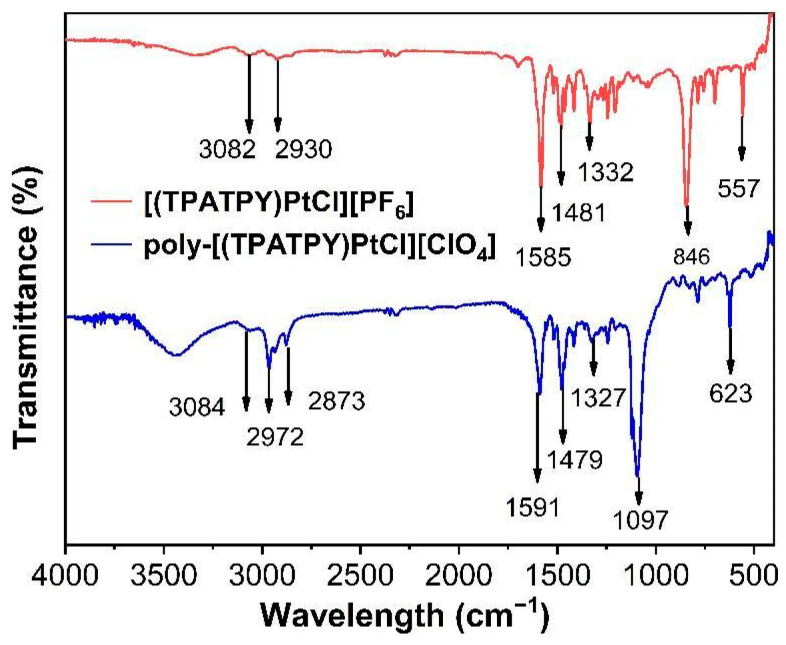
The FT-IR spectra of the complex monomer and the EP film.

The oxidative EP of the target complex on an ITO electrode was also performed for spectroelectrochemical and EC studies. To prove the morphology of the prepared EP film, SEM was employed to examine its surface and cross-section morphologies. As shown in Figure 3, it clearly revealed the connected granular-like surface morphology with typical deposition layers of the EP film after 15 CV cycles, whose thickness was determined to be ca. 290 nm. The qualitative and quantitative elemental compositions of the EP film were further analyzed by an EDS study. The elemental mapping technique exhibited the presence of C, N, O, Cl, and Pt elements (Figure 4). The atomic percentage ratio of Cl to Pt is 2.3:1 (Figure S4 and Table S2), which is close to the ideal value (Cl:Pt = 2:1). The result suggests that the anion PF_6_^−^ of the complex monomer is replaced by the anion ClO_4_^−^ of the supporting electrolyte during the EP process. This is further confirmed by the FT-IR spectrum analysis and the anion-exchange experiment of the EP film. Using stainless steel as the working electrode, the EP film was obtained after 40 CV cycles, which was scraped off of it with a blade. The FT-IR spectra of the EP film sample as well as the complex monomer are shown in Figure 5. Both of them display the C-H vibrations of the benzene ring at ca. 3080 cm^−1^, the C-H vibrations of the pyridine ring at ca. 2900 cm^−1^, the C-C stretching vibrations of the benzene ring at ca. 1590 cm^−1^, the C-N stretching vibrations of the pyridine ring ca. 1480 cm^−1^, and the C-N stretching vibrations of the aromatic tertiary amine of TPA unit at ca. 1330 cm^−1^ [30]. Beside these, the strong P-F stretching vibration at 846 cm^−1^ and the weaker P-F bending vibration at 557 cm^−1^ are present in the FT-IR spectrum of the complex monomer [31], while the peaks at 1097 cm^−1^ and 623 cm^−1^ in the FT-IR spectrum of the EP film sample are attributed to Cl-O stretching and bending vibrations [32], respectively. At the same time, after inserting the EP film into a methanol solution of KPF_6_ and allowing it to stand for 12 h, its color changed significantly (Figure S5). Subsequently, the polymer film was inserted into a methanol solution of LiClO_4_ and left to stand for 12 h, and its color was recovered. This phenomenon indicates that the anions of the EP film do not participate in coordination and can be freely exchanged.

### 2.6. Spectroelectrochemical Performance of Poly-[(L)PtCl][ClO_4_] Film

**Figure 6:**
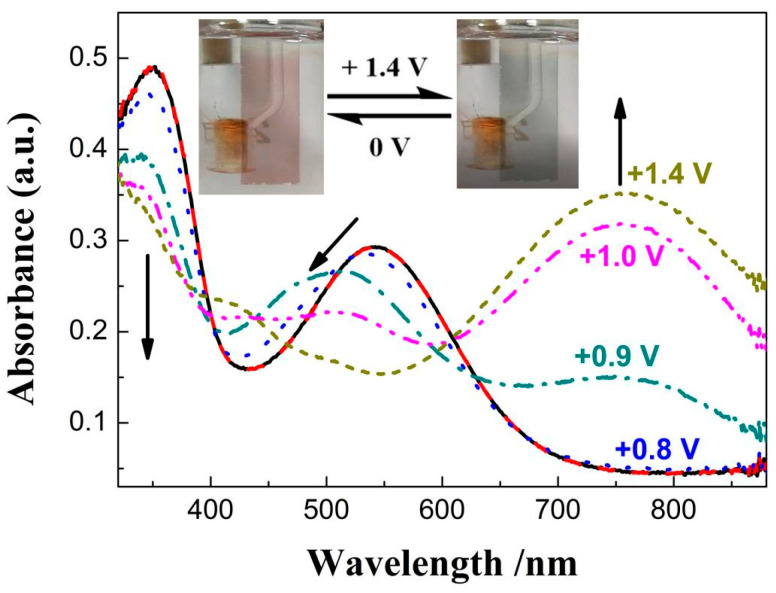
Absorption spectrum changes of the EP film with the applied potentials. Insets: the color changes at 0 V and +1.4 V potentials.

The poly-[(**L**)PtCl][ClO_4_] film (*ca.* 290 nm thickness) coated ITO electrode, prepared by 15 cycles of CV electropolymerization, displayed the significantly red-shifted MLCT/LMCT/LLCT band at *λ*_max_ = 539 nm at the applied potential before +0.9 V (Figure 6). The large difference in the maximum absorption wavelength (Δ*λ*_max_ = 90 nm) between the monomer solution and the metallopolymer film indicates that the more electron-donating segments of N,N,N′,N′-tetraphenyl-4,4′-diaminobiphenyl (TPB) are formed via the electro-cross-linking of the peripheral TPA group in the target complex [12,13,14,15]. When the potential remains below +0.8 V, there is no significant change in the absorption spectrum of this metallopolymer film. If more positive voltage is applied, the ICT characteristic absorption peak is apparently decreased, whereas a broad NIR absorption band centered at *λ*_max_ = 763 nm clearly appears, suggesting the presence of dication (TPB^2+^) species [15]. When the applied voltage is +1.4 V, the NIR band reaches its maximum value and a significant red (CIE coordinates: *L* = 50.75, *a* = 18.58, *b* = 5.69) to dark blue (CIE coordinates: L* = 45.65, a* = −1.35, b* = −12.49) color change is observed in the metallopolymer films. The isosbestic point at *λ* = 609 nm confirms the conversion between the neutral state (TPB) and fully oxidized state (TPB^2+^). On the other hand, the characteristic NIR absorption band of TPB^2+^ species in the neutrally charged poly-[(TPA-C^N^N)PtCl] film is centered at *λ*_max_ = 820 nm [14]. The large difference of the maximum absorption wavelength (Δ*λ*_max_ = 57 nm) between the two systems likely suggests the strong electrostatic interaction of positively charged [(N^N^N)PtCl]^+^ segments limits the delocalization of electrons in this film.

### 2.7. EC Performance of Poly-[(L)PtCl][ClO_4_] Film

**Figure 7:**
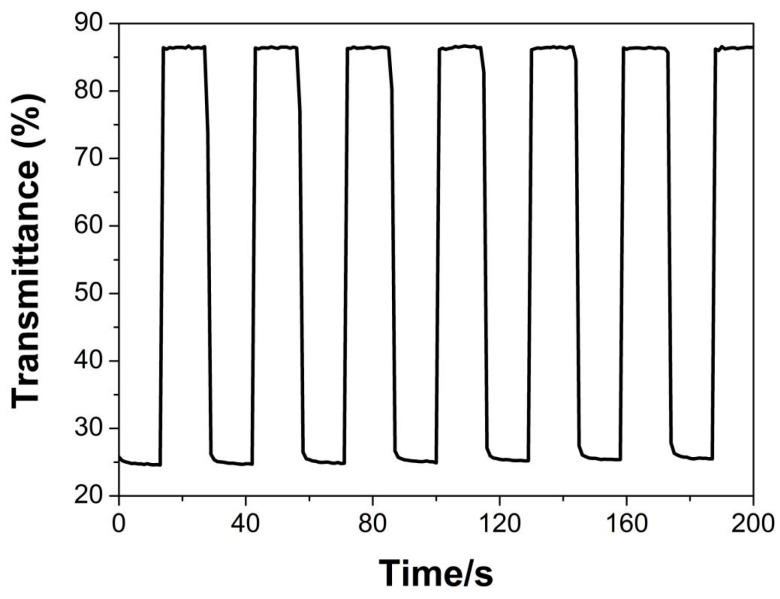
Transmittance changes at 763 nm of the EP film with the step-switching voltage between 0 V and +1.4 V at a time interval of 15 s.

The dynamic EC performance of the metallopolymer film was determined at *λ*_max_ = 763 nm in the blank electrolytic solutions (Figure 7). When the step voltages were applied between 0 V and +1.4 V, the transmittance changed periodically every 15 s. The response times were 2.1 and 0.5 s for the coloring and bleaching steps, respectively, which were determined from the time required for a 95% change of the full transmittance. The optical contrast ratio (Δ*T*%, defined as the transmittance difference between the neutral and oxidative states) of the metallopolymer film was found to be 62%. In addition, the coloration efficiency (CE) was calculated by using the equation: CE = log[(*T*_c_/*T*_b_)/*Q*_d_], where *T*_c_ and *T*_b_ are the transmittances of the oxidative and neutral states, and *Q*_d_ is the injected charge amount per unit area. As a result, the coloring and bleaching efficiencies of this film were 226.5 C^−1^·cm^2^ and 363.0 C^−1^·cm^2^, respectively. This system possesses a slower coloration response but much shorter bleaching time than the parameters (coloration: 1.9 s; bleaching: 2.3 s) of the EP film obtained from the neutrally charged complex [(TPA-C^N^N)PtCl] [14]. These results are likely attributed to the electron-withdrawing effect of the positively charged [(N^N^N)PtCl]^+^ segments, which makes the TPB units in polymer skeleton difficult to oxidize but easy to reduce.

**Figure 8:**
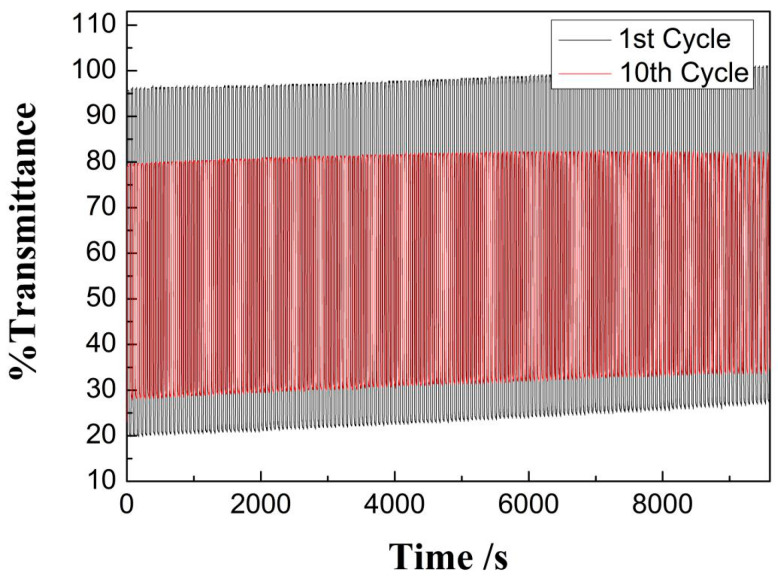
Long duration EC switching testing of the solid-state EC device at 763 nm.

Moreover, a solid-state EC device (ECD) was assembled by using the EP film modified ITO glass working electrode and a blank ITO glass counter electrode and employing homogeneous a PMMA/LiClO_4_/PC/MeCN gel as the electrolyte. After completely gelling for 24 h in a dryer, this ECD demonstrates promising electrochromic performances upon step voltage changes between 0 V and +1.4 V, such as the large optical contrast ratio (Δ*T*%) of 78%, the short response times of 2.9 s for coloration and 1.1 s for bleaching, as well as the high coloration and bleaching efficiency of 278.0 C^−1^·cm^2^ and 390.5 C^−1^·cm^2^, respectively. After 3200 voltage switching cycles, the ECD still maintains 70% of the initial Δ*T*% value (Figure 8), suggesting the solid-state ECD has much longer stability than that of this film in an electrolytic solution. The EC performances of other metallopolymer films based on Ru(II)- and Fe(II)-terpyridine complexes are also listed in Table S3. Because their solid-state devices were rarely reported, it is difficult to accurately judge the EC performance of this system through comparison. But overall, the three key parameters of this system, namely the response time, the optical contrast ratio, and the cycling stability, are significantly better than those of other systems.

## 3. Materials and Methods

### 3.1. Materials

The analytical grades of reagents 2-acetylpyridine, 4-bromophenylaldehyde, diphenylamine, 18-crown-6, polymethyl methacrylate (PMMA), propylene carbonate (PC), anhydrous K_2_CO_3_, K_2_PtCl_4_, CuI, and LiClO_4_ were commercially available and employed without additional purification. Indium tin oxide (ITO) conducted glass (10 Ω/square) was provided by Shenzhen Nanbo Display Technology Co. LTD., and carefully cleaned before use [14]. The target ligand, 4′-[(p-(diphenylamino)phenyl)]-2,2′:6′,2″-terpyridine (L), was prepared using the previously published methodology [29]. *n*-Tetrabutylammonium perchlorate (*^n^*Bu_4_NClO_4_) supporting electrolyte was homemade in our lab and used in electrochemical experiments [15] (Caution! It is important to handle perchlorate salts carefully and in tiny amounts to avoid explosions.).

### 3.2. Synthesis of the [(L)PtCl]·PF_6_ Complex

**L** (0.48 g, 1.0 mmol) and the metal salt K_2_PtCl_4_ (0.45 g, 1.1 mmol) were dissolved in a mixed solvent (100 mL) with equal volume of CH_3_CN and H_2_O. For three days, the suspension was refluxed in an atmosphere of Ar. Aqueous NH_4_PF_6_ was added once it had reached room temperature, and the resulting precipitate was further separated by silica gel columns with CH_3_CN/saturated aqueous KNO_3_/H_2_O as an eluent. The predominant red fraction was collected and halved in volume. After being treated with aqueous NH_4_PF_6_, the resulting precipitate was filtered out and then washed with water and ether before being dried under vacuum for 24 h. An aqueous solution of NH_4_PF_6_ was used to treat the resulting precipitate, which was then filtered and cleaned by water and ether washing. After vacuum drying for 24 h, the target complex was produced as red powder (yield: 65%). ^1^H NMR (400 MHz, DMSO-*d*_6_): *δ* 8.73–8.67 (m, 6H), 8.45 (t, *J* = 8.0 Hz, 2H), 7.97 (d, *J* = 8.4 Hz, 2H), 7.85 (t, *J* = 6.4 Hz, 2H), 7.44 (t, *J* = 7.6 Hz, 4H), 7.26–7.18 (m, 6H), 7.05 (d, *J* = 8.8 Hz, 2H). ^13^C NMR (100 MHz, DMSO-*d*_6_): *δ* 158.9, 154.4, 152.4, 151.6, 150.9, 146.4, 142.9, 130.4, 129.8, 129.5, 127.0, 126.3, 126.1, 125.4, 120.7, 120.4. Anal. Calcd (%): C, 49.14; H, 3.00; N, 6.95. Found: C, 48.91; H, 2.94; N, 6.77.

### 3.3. Characterization

A Bruker AV 400 spectrometer was used for the ^1^H and ^13^C NMR measurements and referenced against an internal standard of tetramethylsilane (TMS) and Si(CH_3_)_4_. Elemental analysis experiments were performed using a Bio-Rad Co elemental analytical device. Absorption spectroscopic measurements were conducted with a HITACHI UH4150 spectrophotometer for UV, visible, and near-infrared absorption. The electrochemical workstation CHI660A (CH Instruments Co., Shanghai, China) was used to perform the cyclic voltammetry (CV) studies. Using ferrocene as a redox probe, in a 0.1 mol·dm^−3^ *^n^*Bu_4_NClO_4_/CH_3_CN solution with 1.0 × 10^−3^ mol·dm^−3^, the AC impedance experiments were conducted by varying the frequency between 1 and 100 kHz with an AC voltage of +0.50 V vs. SCE. Morphology and elemental analyses were performed using a Carl Zeiss Sigma 500 Field-emission scanning electron microscope (FE-SEM) equipped with an energy dispersive spectrometer (EDS). The FT-IR spectra were measured via a Shimadzu IRTracer-100 spectrometer using KBr pellets. The CIE L*, a* and b* values of the samples were measured via a WR-10QC Colorimeter (Shenzhen, China).

### 3.4. X-Ray Crystallography

In order to determine the structure of target ligand **L**, a proper single crystal of it (0.16 mm × 0.14 mm × 0.11 mm) was employed. A Bruker SMART APEX-II CCD diffractometer was utilized in the *Φ*-*ω* scan mode to produce the crystal diffraction data, employing graphite-monochromated Mo-*Kα* radiation (*λ* = 0.71073 Å) at a temperature of 296(2) K. Within the specified range of 2.30 < q < 24.99°, with −10 ≤ h ≤ 9, −11 ≤ k ≤ 11, and −34 ≤ l ≤ 23, 12,797 reflections were harvested. Of these, 4377 unique reflections were involved (*R*_int_ = 0.0469). Using the SADABS mode, an absorption correction was implemented [33]. SHELX-97 software was used to directly solve the structure and refine it via the full-matrix least-squares method on *F*^2^ [34]. Anisotropic refinement of non-hydrogen atoms, and isotropic refinement of hydrogen atoms were employed here, accompanied by a geometrical generation of H atoms.

### 3.5. Preparation of the Hybrid Metallopolymer Film

The hybrid metallopolymer film was prepared using the oxidative electropolymerization (EP) method. Using a CHI 660A electrochemical workstation, the electrochemical deposition of film on the working Pt disk or ITO glass was performed in a 0.1 mol·L^−1^ *^n^*Bu_4_NClO_4_/CH_3_CN solution with 2.5 × 10^−4^ mol·L^−1^ [(**L**)PtCl][PF_6_] monomer using cyclic voltammetry at scan rate of 50 mV·s^−1^. A three-electrode system employing an ITO glass (ca. 0.7 cm × 5 cm) working electrode, a platinum wire counter electrode, and an Ag wire or a saturated calomel electrode (SCE) as the reference electrode, was adopted here. A thorough rinsing of the EP film in CH_3_CN solvent was conducted to wash off any unreacted monomer or small oligomers.

### 3.6. Spectroelectrochemical and EC Characterization

Similar instrumental methods to those that were previously published [15] were used to evaluate the spectroelectrochemical and EC performance of the hybrid metallopolymer film coated ITO glass in a 0.1 mol·L^−1 *n*^Bu_4_NClO_4_/CH_3_CN solution. Briefly, the ITO electrode coated with EP film was placed in a quartz cell containing a Pt wire counter electrode and an Ag wire reference electrode. The absorption spectra were then recorded using a HITACHI UH4150 spectrophotometer, with the potentials being controlled by a CHI660A electrochemical workstation. To study the electrochromic performances, a double potential step chronoamperometry method was employed, and the transmittance curve at characteristic wavelengths was recorded in real time between the neutral and oxidized states.

### 3.7. EC Solid-State Device Fabrication and Characterization

Similar procedures [15] were applied to fabricate the sandwich-type test devices, but the PMMA/LiClO_4_/PC/MeCN gel electrolyte was implemented instead [35]. Briefly, the solid-state EC device (ECD) was prepared employing the EP film modified ITO conductive glass (size: 2 cm × 4 cm) with a coverage area of 1 cm × 2 cm as the working electrode, a bare ITO glass (size: 3 cm × 3 cm) as the counter electrode and the homogeneous PMMA/LiClO_4_/PC/MeCN gel as the electrolyte. Upon spin-coating the gel electrolyte onto the working electrode at a rate of 500 rpm, the resulting working electrode and the counter electrode were stacked face-to-face with clips. Prior to testing, the devices were placed in a desiccator for 24 h to ensure complete gelation. For EC performance test, the transmission spectra were in situ measured as functions of the applied double-step potential utilizing the EP film-free blank solid-state device as a reference.

## 4. Conclusions

In conclusion, a conductive metallopolymer film possessing a diffusion controlled redox nature was fabricated via an efficient TPA-radical-triggered polymerization mechanism from a novel TPA functionalized [(N^N^N)PtCl][PF_6_] complex. Its morphology, thickness, elemental composition, and anion-exchange effect were further characterized by SEM, EDS, and FT-IR methods. The hybrid film showed the near-IR EC effect with notable color switching from red to dark blue upon oxidation. The good EC performance of its solid-state device, such as the large optical contrast (Δ*T*%) of 78%, the fast response times of 2.9 s for coloration and 1.1 s for bleaching, the high coloration efficiency of 278.0 C^−1^·cm^2^ and bleaching efficiency of 390.5 C^−1^·cm^2^, and long-term stability, suggests the promising application of this hybrid metallopolymer film in information and image display. At the same time, with reference to the behaviors of the EP films formed from polypyridine Pt(II) and Ru(II) complexes, the coordination configuration and electronic structure of the metal complex were verified to be two key factors for designing EC materials with superior performance.

## Data Availability

The data presented in this study are available on request from the corresponding author.

## Supplementary Materials

The following supporting information can be downloaded at: https://www.mdpi.com/article/10.3390/molecules28248027/s1, Figure S1: ^1^H NMR spectrum of complex [(**L**)PtCl][PF_6_]; Table S1: Crystal data and crystal structure parameters of **L**; Figure S2: Crystal packing of ligand **L**; Figure S3: UV-vis absorption spectra of the target ligand **L** and complex [(**L**)PtCl][PF_6_] in CH_3_CN solution; Figure S4: The element analysis diagram of the EP film coated ITO electrode; Table S2: The element analysis result of the EP film coated ITO electrode; Figure S5: The color change of the EP film after the ion-exchange effect; Table S3: The EC performance of the TPY-based metallopolymer films in solution or solid-state device. References [36,37,38,39,40,41,42,43].
