# Supplementary material for: Near-IR Electrochromic Film with High Optical Contrast and Stability Prepared by Oxidative Electropolymerization of Triphenylamine Modified Terpyridine Platinum(II) Chloride
Source: Molecules. 2023 Dec 9;28(24):8027. doi: 10.3390/molecules28248027 (PMC10745481; doi:10.3390/molecules28248027)
Supplement: Supplementary file 1 [file molecules-28-08027-s001.zip › molecules-2719706-supplementary.pdf]

Supplementary Materials

Near-IR Electrochromic Film with High Optical Contrast and Stability Prepared by Oxidative Electropolymerization of Triphenylamine Modified Terpyridine Platinum(II) Chloride

Huiying Gu [1,†], Xiaomeng Sun [2,†], Qian Zhao [2], Hongwei Wang [2], Xinfeng Cheng [2,*], Chunxia Yang [2], Dongfang Qiu [2,*]

1 College of Chemistry, Zhengzhou University, No. 100 of Kexue Road, Zhengzhou 450001, China

2 College of Chemistry and Pharmaceutical Engineering, Nanyang Normal University, Nanyang 473061, China

* Correspondence: x.f.cheng@nynu.edu.cn (X.C.); qiudf2008@nynu.edu.cn (D.Q.)

† These authors contributed equally to this work.

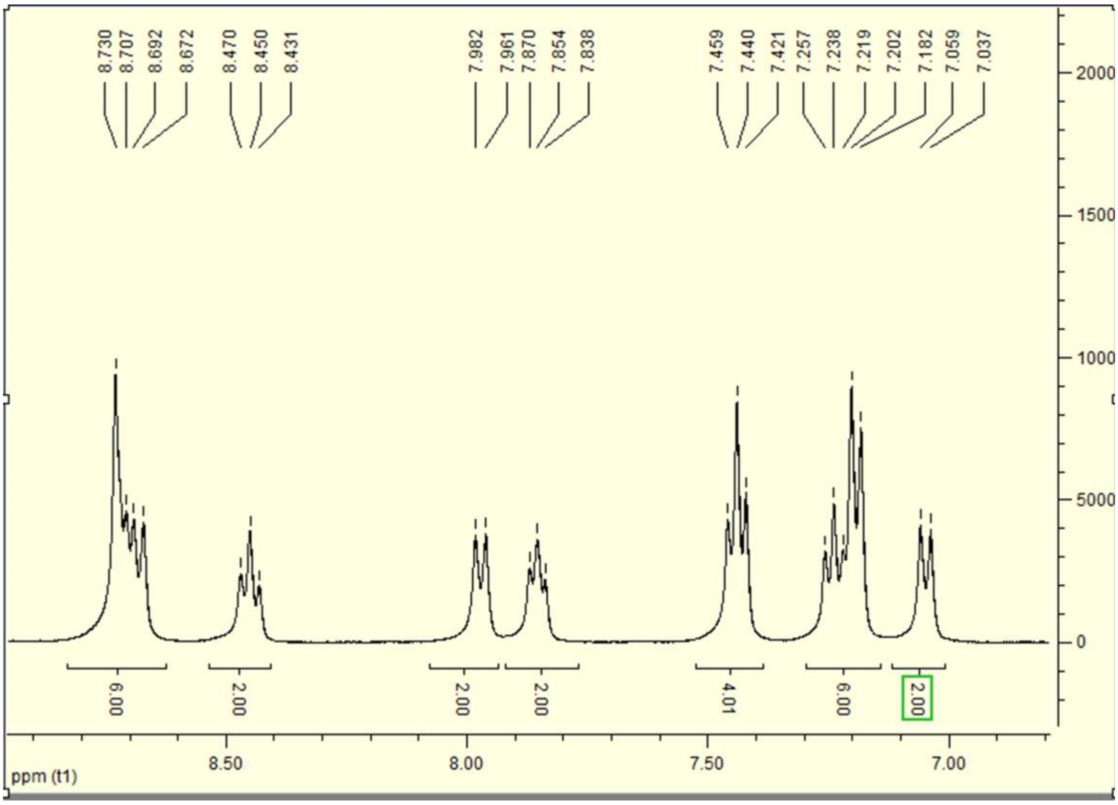

Figure S1. ^{1}H NMR spectrum of complex [(**L**)PtCl][PF_6].

Table S1. Crystal data and crystal structure parameters of **L**.

**Compound**	**L**
Empirical formula	$C_{33}H_{24}N_4$
Formula weight	476.56
Temperature/ K	296(2)
Wavelength/Å	0.71073
Crystal system	Orthorhombic
Space group	$P2_12_12_1$
a / Å	9.102(4)
b / Å	9.307(4)
c / Å	29.371(11)
α / (°)	90.00
β / (°)	90.00
γ / (°)	90.00
Volume / Å^3	2488.1(17)
Z	4
D_c / (g·cm^{-3})	1.272
Absorption coefficient / mm^{-1}	0.076

$F(000)$	1000
Crystal size / mm	0.16×0.14×0.11
θ range for data collection /(°)	2.30 to 24.99
Limiting indices	$-10 \leq h \leq 9$, $-11 \leq k \leq 11$, $-34 \leq l \leq 23$
Completeness to θ = 25.00°	99.9 %
Data / restraints / parameters	4377/1/335
Goodness-of-fit on F^2	1.104
Final R indices [$I > 2\sigma(I)$]	R_1 = 0.0431, wR_2 = 0.0685
R indices (all data)	R_1 = 0.0804, wR_2 = 0.0760
Largest diff. peak and hole/(e.Å^{-3})	0.138 and -0.115

[a] $R_1=\sum||F_0|-|F_c||/\sum|F_0|$, $wR_2=\{\sum w[(F_0)^2-(F_c)^2]^2/\sum w[(F_0)^2]^2\}^{1/2}$

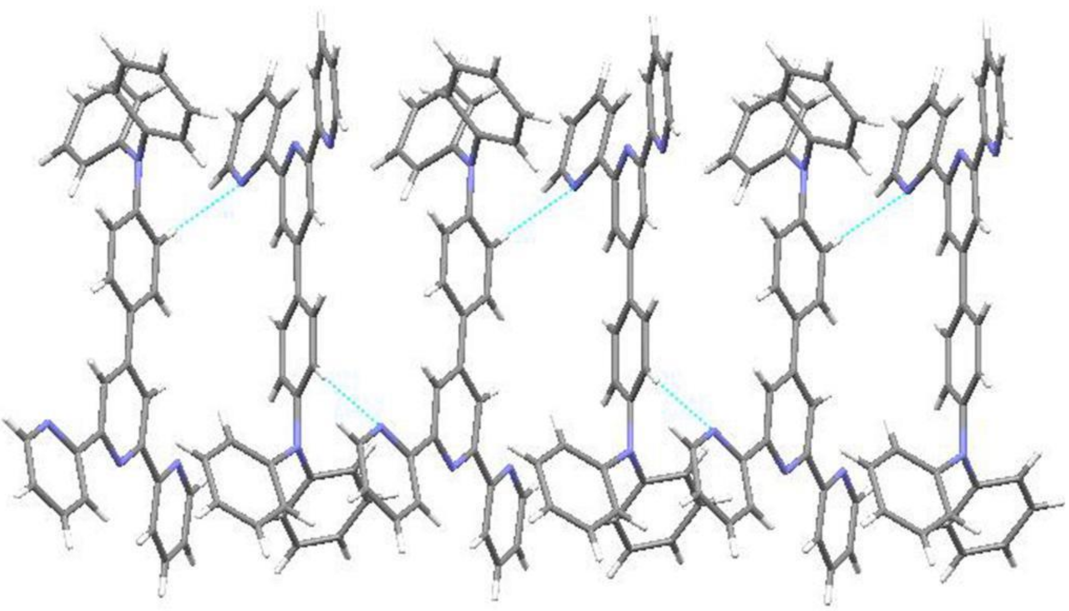

Figure S2. Crystal packing of ligand **L**.

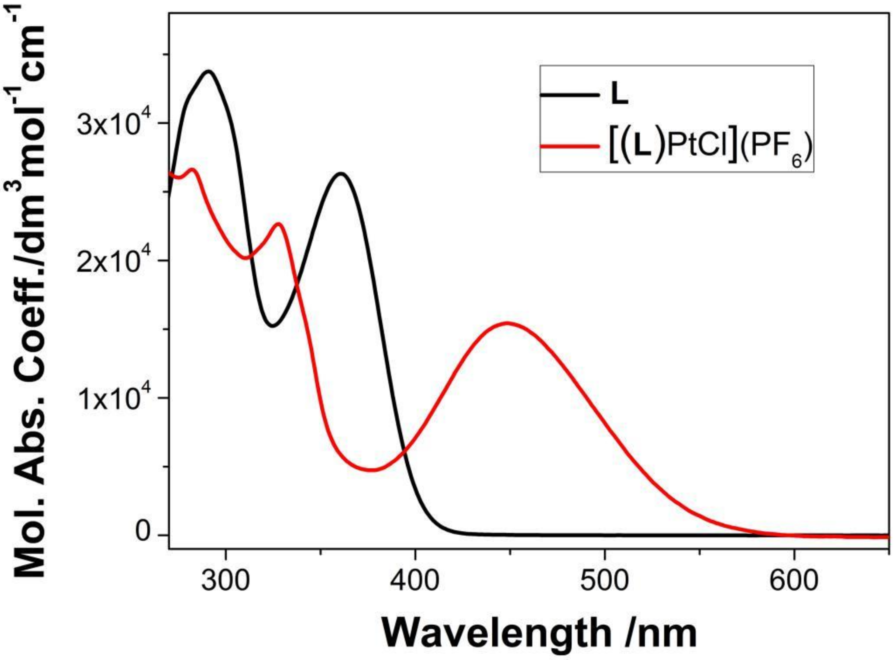

Figure S3. UV-vis absorption spectra of the target ligand **L** and complex [(**L**)PtCl][PF_6] in CH_3CN solution.

.

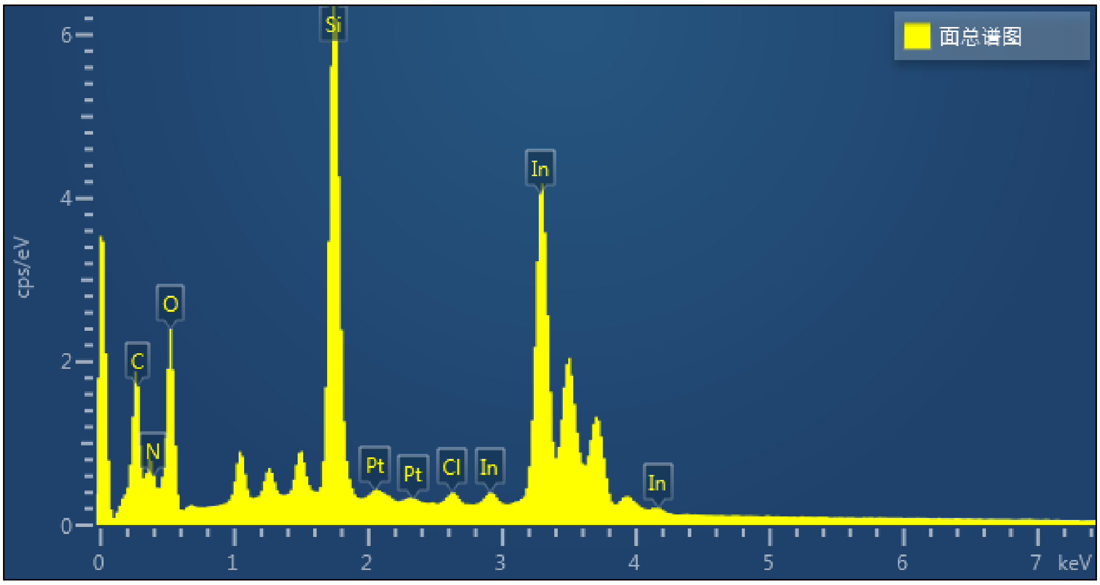

Figure S4. The element analysis diagram of the EP film coated ITO electrode.

Table S2. The element analysis result of the EP film coated ITO electrode.

Element	X-ray series	wt%	Atomic percentage (%)
C	K	13.89	32.18
N	K	2.39	4.75
O	K	20.58	35.80
Si	K	15.78	15.64
Cl	K	0.50	0.40
In	L	45.69	11.07
Pt	M	1.18	0.17
total:		100.00	100.00

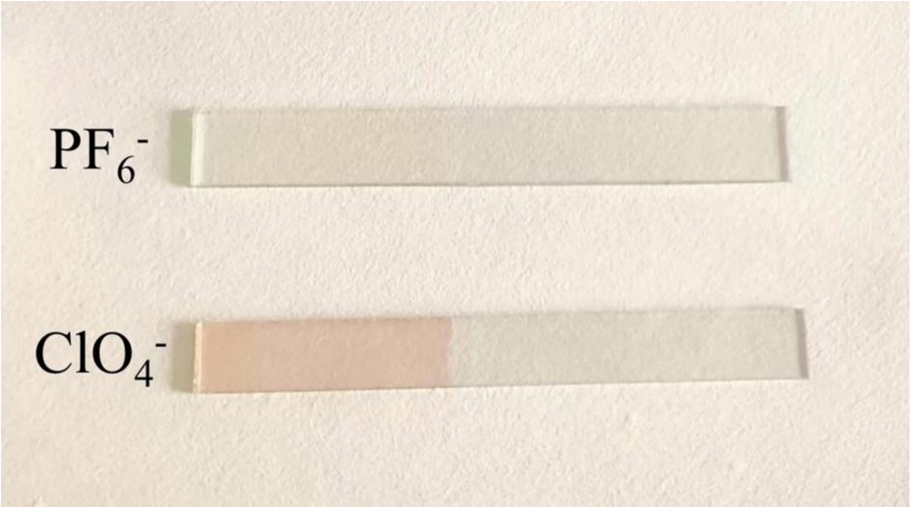

Figure S5. The color change of the EP film after the ion-exchange effect.

Table S3. The EC performance of the TPY-based metallopolymer films in solution or solid-state device.

<table>
<tr><th rowspan="3">Preparation Method</th><th rowspan="3">Ligand Structure</th><th rowspan="3">Metal ions</th><th rowspan="3">ECD type</th><th colspan="8">EC performance</th><th rowspan="3">Reference</th></tr>
<tr><th rowspan="2">λ_{max} (nm)</th><th rowspan="2">Color Change</th><th colspan="2">Coloration Step</th><th colspan="2">Bleaching Step</th><th rowspan="2">ΔT_{max} (%)</th><th rowspan="2">Long term stability</th></tr>
<tr><th>Response time (s)</th><th>CE ($C^{-1}\cdot cm^2$)</th><th>Response time (s)</th><th>CE ($C^{-1}\cdot cm^2$)</th></tr>
<tr><td rowspan="9">**Electropolymerization (EP)**</td><td rowspan="3">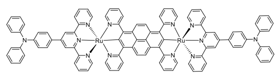</td><td rowspan="6">Ru(II)</td><td rowspan="6">Liquid</td><td>2150</td><td rowspan="2">dark blue / brown / orange</td><td>15-20</td><td>550</td><td>15-20</td><td>-</td><td>50</td><td rowspan="6">-</td><td rowspan="6">1</td></tr>
<tr><td rowspan="2">1500</td><td rowspan="2">decades</td><td rowspan="2">100</td><td rowspan="2">decades</td><td rowspan="2">-</td><td>27</td></tr>
<tr><td>23</td></tr>
<tr><td rowspan="3">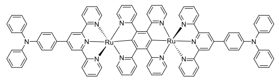</td><td rowspan="3">1185</td><td rowspan="3">blue / brown/olive /green</td><td rowspan="3">15-20</td><td rowspan="3">220</td><td rowspan="3">-</td><td rowspan="3">-</td><td>28</td></tr>
<tr><td>22</td></tr>
<tr><td>35</td></tr>
<tr><td rowspan="2">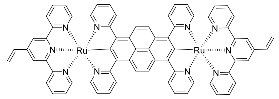</td><td rowspan="2">Ru(II)</td><td rowspan="2">Liquid</td><td rowspan="2">2050</td><td rowspan="2">Blue/brown/orange</td><td>6</td><td>220</td><td>2</td><td>-</td><td>35</td><td>100</td><td rowspan="2">2</td></tr>
<tr><td>2</td><td>-</td><td>6</td><td>-</td><td>32</td><td>-</td></tr>
<tr><td>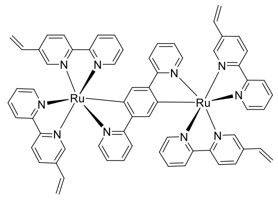</td><td>Ru(II)</td><td>Liquid</td><td>1300</td><td>Wine/orange/dark cyan</td><td>15-20</td><td>200</td><td>-</td><td>-</td><td>41</td><td>30</td><td>3</td></tr>
</table>

	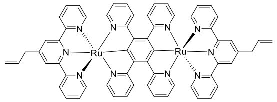	Ru(II)	Liquid	1165	Blue/pink/green	6	250	5	-	40	-	4
						4	-	8	-	37	-	
	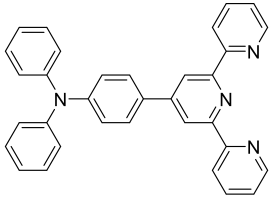	Pt(II)	Liquid	763	Red/dark blue	1.9	226.5	2.3	363.0	62	-	this work
			Solid			2.9	278.0	1.1	390.5	78	3200	
**Lay-by-layer (LBL) assembly**	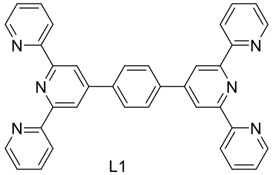 L1	Fe(II)	Liquid	580	Blue/colorless	0.31	263.8	0.58	-	41.6	-	5
	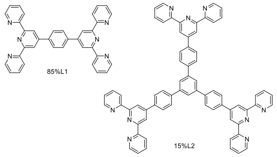 85%L1 15%L2					0.19	383.4	0.36	-	50.7	-	
	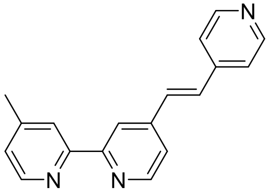	Fe(II)	Liquid	575	Purple/colorless	0.4	955	-	-	41	1000	6

	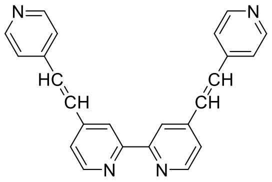			591	Bluish gray/ colorless	0.3	1488	-	-	31	1000	
	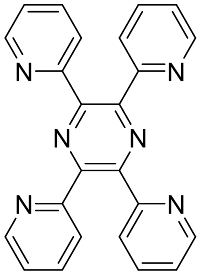	Fe(II)	Solid	630	bluish /transparent	620ms	70	-	-	70	103	7
	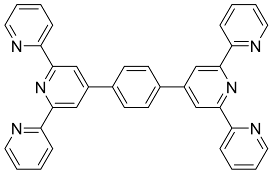	Fe(II)	Liquid	594	Blue/colorless	several	500	-	-	-	1000	8
**Liquid-liquid interfacial self-assembly**	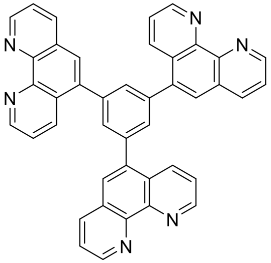	Fe(II)	Liquid	518	Red/colorless	3.3	230	2.9	-	56	-	9
			Solid			9	-	6	-	65	15000	

	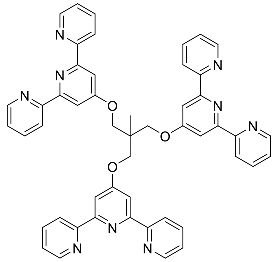	Fe(II)	Liquid	556	Intense pink/colorless	0.84	-	0.88	-	-	-	10
			Solid			1.15	470.16	2.49	-	53	1000	
	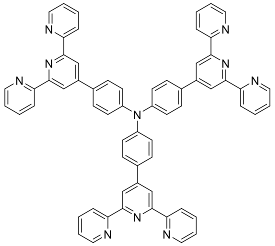	Fe(II)	Liquid	580	purplish red /orange-yellow /green	0.5	141.72	0.4	-	22.3	500	11
			Solid			1	-	0.9	-	-	-	
	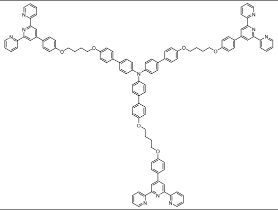	Fe(II)	Liquid	570	Purple/yellow-green	14.3	172.82	7.3	-	20	-	12

Reference

[1] Chang-Jiang Yao, Yu-Wu Zhong, and Jiannian Yao, Five-Stage Near-Infrared Electrochromism in Electropolymerized Films Composed of Alternating Cyclometalated Bisruthenium and Bis-triarylamine Segments, *Inorg. Chem.* 2013, 52, 10000-10008.

[2] Chang-Jiang Yao, Jiannian Yao, and Yu-Wu Zhong, Metallopolymeric Films Based on a Biscyclometalated Ruthenium Complex Bridged by 1,3,6,8-Tetra(2-pyridyl)pyrene: Applications in Near-Infrared Electrochromic Windows, *Inorg. Chem.* 2012, 51, 6259-6263.

[3] Hai-Jing Nie and Yu-Wu Zhong, Near-Infrared Electrochromism in Electropolymerized Metallopolymeric Films of a Phen-1,4-diyl-Bridged Diruthenium Complex, *Inorg. Chem.* 2014, 53, 11316–11322.

[4] Chang-Jiang Yao, Yu-Wu Zhong, Hai-Jing Nie, Héctor D. Abruña, and Jiannian Yao, Near-IR Electrochromism in Electropolymerized Films of a Biscyclometalated Ruthenium Complex Bridged by 1,2,4,5-Tetra(2-pyridyl)benzene, *J. Am. Chem. Soc*. 2011, 133, 20720-20723.

[5] Chih-Wei Hu, Takashi Sato, Jian Zhang, Satoshi Moriyama, and Masayoshi Higuchi, Three-Dimensional Fe(II)-based Metallo-Supramolecular Polymers with Electrochromic Properties of Quick Switching, Large Contrast, and High Coloration Efficiency, *ACS Appl. Mater. Interfaces* 2014, 6, 9118-9125

[6] Sreejith Shankar, Michal Lahav, and Milko E. van der Boom, Coordination-Based Molecular Assemblies as Electrochromic Materials: Ultra-High Switching Stability and Coloration Efficiencies, *J. Am. Chem. Soc.* 2015, 137, 4050-4053.

[7] Clóvis A. da Silva, Marcio Vidotti, Pablo A. Fiorito, Susana I. Córdoba de Torresi, Roberto M. Torresi, and Wendel A. Alves, Electrochromic Properties of a Metallo-supramolecular Polymer Derived from Tetra(2-pyridyl-1,4-pyrazine) Ligands Integrated in Thin Multilayer Films, *Langmuir* 2012, 28, 3332-3337.

[8] Marco Schott, Wojciech Szczerba, and Dirk G. Kurth, Detailed Study of Layer-by-Layer Self-Assembled and Dip-Coated Electrochromic Thin Films Based on Metallo-Supramolecular Polymers, *Langmuir* 2014, 30, 10721-10727.

[9] Sanjoy Mondal, Yoshikazu Ninomiya, Takefumi Yoshida, Taizo Mori, Manas Kumar Bera, Katsuhiko Ariga, and Masayoshi Higuchi, Dual-Branched Dense Hexagonal Fe(II)-Based

Coordination Nanosheets with Red-to-Colorless Electrochromism and Durable Device Fabrication, *ACS Appl. Mater. Interfaces* 2020, 12, 31896-31903.

[10] Susmita Roy and Chanchal Chakraborty, Interfacial Coordination Nanosheet Based on Nonconjugated Three-Arm Terpyridine: A Highly Color-Efficient Electrochromic Material to Converge Fast Switching with Long Optical Memory, *ACS Appl. Mater. Interfaces* 2020, 12, 35181-35192.

[11] Yu Kuai, Weijun Li, Yujie Dong, Wai-Yeung Wong, Shuanma Yan, Yuyu Daia and Cheng Zhang, Correction: Multi-color electrochromism from coordination nanosheets based on a terpyridine-Fe(ii) complex Check for updates, *Dalton Trans.*, 2019, 48, 16458-16458.

[12] Yu Kuai, Tao Yang, Feiya Yuan, Yujie Dong, Qingbao Song, Cheng Zhang, Wai-Yeung Wong, Self-assembled flexible metallo-supramolecular film based on Fe(II) ion and triphenylamine-subsituted alkyl terpyridine towards electrochromic application, *Dyes and Pigments*, 2021, 194, 109623.
